# GPR68 limits the severity of chemical-induced oral epithelial dysplasia

**DOI:** 10.1038/s41598-023-27546-y

**Published:** 2023-01-07

**Authors:** David Shore, Nosakhere Griggs, Vincent Graffeo, A. R. M. Ruhul Amin, Xiang-ming Zha, Yan Xu, Jeremy P. McAleer

**Affiliations:** 1 Marshall University School of Pharmacy, Huntington, WV USA; 2 Marshall University Joan C. Edwards School of Medicine, Huntington, WV USA; 3 University of Missouri-Kansas City School of Pharmacy, Kansas City, MO USA; 4 Indiana University School of Medicine, Indianapolis, IN USA

**Keywords:** Head and neck cancer, Oral cancer

## Abstract

Head and neck cancer is the sixth most common malignancy, and there is an urgent need to identify physiological processes contributing to tumorigenesis. Extracellular acidification caused by aerobic glycolysis within tumor microenvironments can stimulate proton-sensing receptors. GPR68, or ovarian cancer G protein-coupled receptor 1, responds to extracellular acidity and is highly expressed in head and neck squamous cell carcinoma (HNSCC) as well as normal esophageal tissue. To study the role of GPR68 in oral dysplasia, wild-type and GPR68^−/−^ mice were treated with 4-Nitroquinoline N-oxide (4NQO) in drinking water for 11–13 weeks, followed by normal water for 11–12 weeks. 4NQO treatment resulted in 45 percent of GPR68^−/−^ mice developing severe dysplasia or squamous cell carcinoma compared to only 10.5 percent of GPR68^+/+^ mice. This correlated with increased frequencies of regulatory T cells in the spleens of male GPR68^−/−^ mice. Dysplastic regions of the tongue had increased CD31 staining compared to normal regions in both GPR68^−/−^ and GPR68^+/+^ mice, suggesting that angiogenesis was GPR68-independent. RNA knockdown studies using HNSCC cell lines demonstrated no direct effect of GPR68 on survival or growth. Overall, we demonstrate that GPR68-deficiency worsens the severity of chemical-induced oral dysplasia, suggesting a protective role for this gene in tumorigenesis.

## Introduction

Head and neck cancer is the sixth most common cancer worldwide and contributes to more than 66,000 new cases and 15,000 deaths in the United States each year^1^. Arising in the epithelial layers of the oral cavity, pharynx, and larynx, 90 percent of head and neck cancer cases are squamous cell carcinomas (HNSCC)^2^. Major risk factors include tobacco and alcohol use, as well as Human Papilloma Virus infection. Even after improvements in traditional treatments including chemotherapy, radiotherapy and surgery, the prognosis has remained unchanged for several decades with 5-year survival rates below 50 percent for patients with advanced stage disease. The pathophysiology of HNSCC involves a series of genetic alterations affecting tumor suppressors and oncogenes within epithelial mucosa, such as p53, EGFR, STAT3 and VEGFR^2^. This leads to hyperplasia, followed by oral epithelial dysplasia (OED) that progresses from mild to severe, then carcinoma in situ and ultimately invasive carcinoma and metastatic disease. OED severity positively correlates with progression to invasive carcinoma, as 2.6% of mild, 4.1% of moderate and 29.2% of severe OED cases progressed to HNSCC^3^. With no reliable biomarkers for OED progression, diagnosis is through biopsies that are evaluated for architectural and cytological alterations in the surface epithelium^4^. Identifying factors that contribute to OED development and progression may aid in designing preventative treatments for HNSCC, as early diagnosis significantly improves patient outcomes and survival^5^.

The tumor microenvironment is constructed during the course of tumorigenesis by the collection of cell types present, setting up conditions that facilitate cancer development^6^. Aerobic glycolysis is a near-universal trait of cancer and leads to extracellular acidosis^7^. The extent to which acidosis induces tumorigenesis or results from tumorigenesis has not been completely elucidated; however, extracellular acidification can regulate proliferation, survival, metastasis, inflammation, anti-tumor immunity and angiogenesis^7–9^.

GPR68, also known as ovarian cancer G protein-coupled receptor 1 (OGR1), is a proton-sensing G-protein-coupled receptor that responds to extracellular acidity^10^. GPR68 is expressed in several tissues such as the esophagus, stomach, intestine, bone, endothelium, immune system, brain^11^, lungs and cancer. Tumor xenograft models have demonstrated that GPR68^-/-^ mice are protected from tumorigenesis, suggesting that its expression in hematopoietic cells promotes the growth of subcutaneously injected tumors^12,13^. The role of GPR68 in HNSCC is unknown; however, gene expression data shows that HNSCC expresses the highest levels of GPR68 among solid tumors, correlating with high expression in normal esophageal tissue^10^. Despite these data, the role of endogenous GPR68 expression on OED or oral tumorigenesis has not been assessed.

Here, we studied the impact of GPR68-deficiency on susceptibility to chemical-induced OED and SCC using a murine model that recapitulates the development and progression of oral lesions associated with tobacco use. 4-Nitroquinoline N-oxide (4NQO) is an aromatic heterocyclic compound that forms DNA adducts by preferentially binding to guanine residues, resulting in pyrimidine substitutions within genes involved in carcinogenesis^14^. In addition, 4NQO induces oxidative stress which further promotes DNA damage^15^. We found that treatment with 4NQO resulted in similar numbers of tongue lesions between wild-type (GPR68^+/+^) and GPR68^−/−^ mice; however, GPR68^−/−^ mice were more likely to develop severe dysplasia and SCC. This correlated with significantly increased frequencies of regulatory T cells (Tregs) in the spleens of male GPR68^−/−^ mice. In vitro experiments revealed that GPR68 expression did not directly affect Treg differentiation, suggesting that T cell-extrinsic expression of GPR68 may regulate CD4 T cell responses in vivo. Dysplastic regions of the tongue had significantly elevated expression of the angiogenesis marker CD31 compared to normal regions in both mouse strains, suggesting that angiogenesis induced by 4NQO treatment was GPR68-independent. RNA knockdown studies revealed that GPR68 did not directly affect the survival and growth of head and neck cancer cell lines. Overall, we demonstrate that endogenous GPR68 expression limits the severity of chemical-induced OED in a tumor-extrinsic manner, suggesting a protective role in carcinogenesis.

## Results

### GPR68 deficiency increases the severity of chemical-induced oral epithelial dysplasia (OED)

**Figure 1:**
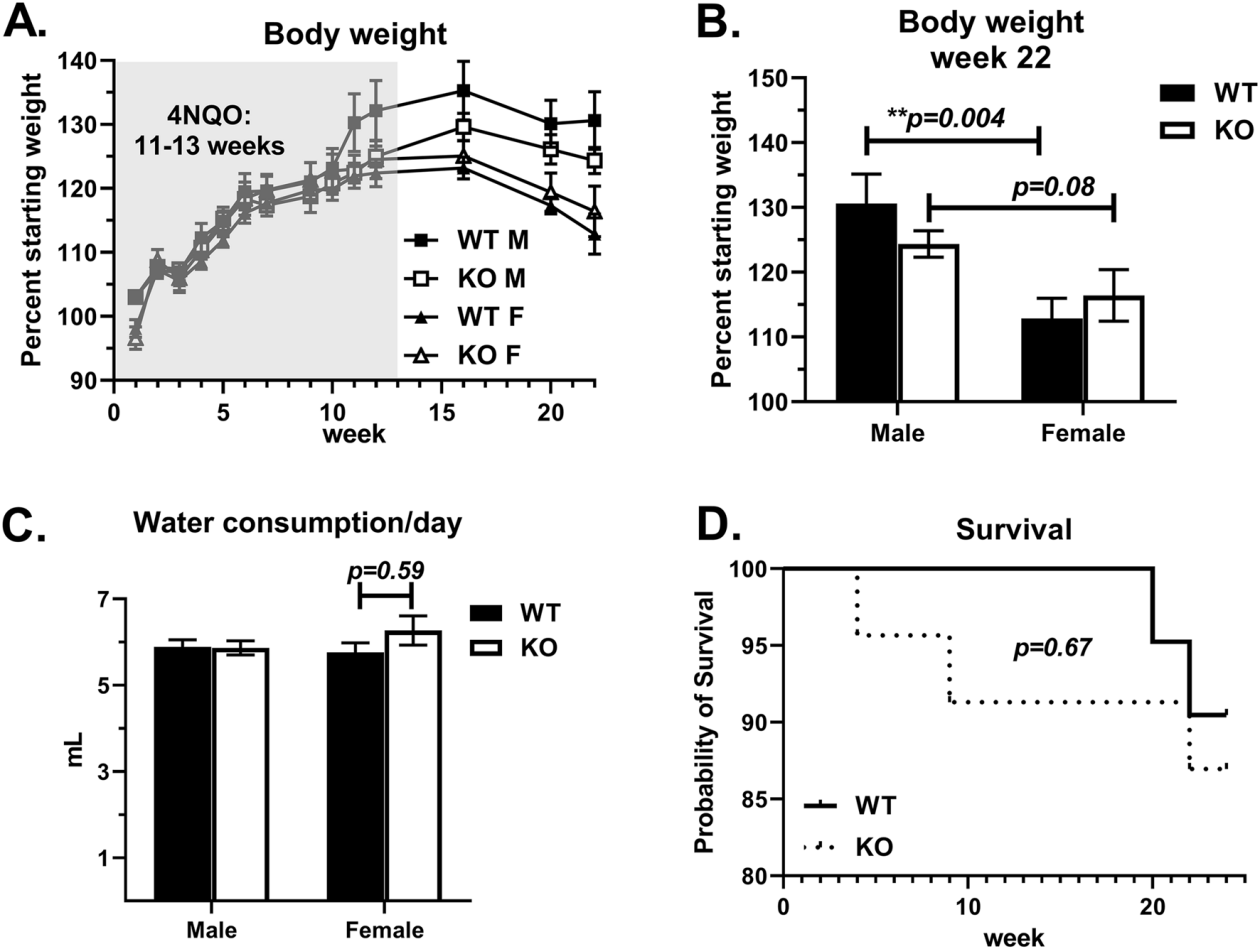
Endogenous GPR68 expression does not significantly affect body weight, water consumption or survival following oral 4-Nitroquinoline N-oxide (4NQO) treatment. WT (closed symbols) and KO (open symbols) mice were separated into male (M) and female (F) groups and placed on 4NQO drinking water (0.05 mg/mL) for 11–13 weeks, followed by normal water for 11–12 weeks. (**A**) Body weight reported as a percent of starting weight (week 0 = 100%) at the indicated timepoints. Area under the curve analysis revealed no differences between the groups. (**B**) Body weight at week 22. (**C**) Average amount of 4NQO water consumed per mouse each day. Water was replaced each week, with the volume consumed divided by the number of mice in each cage. This number was then divided by the days since the last water change. (**D**) Time course showing the percent of WT (solid line) or KO (dotted line) mice surviving at each weekly timepoint. Data are combined from three independent experiments and shown as the mean +/− SEM (WT M n = 9; WT F n = 10; KO M n = 11, KO F n = 9). Asterisks indicate statistically significant differences between the groups (**P < 0.01).

Administering 4NQO in murine drinking water results oral epithelial mutagenesis through similar mechanisms as the carcinogens in tobacco products. The stepwise progression from oral epithelial hyperplasia to dysplasia and SCC is similar to that observed in human patients, making 4NQO a suitable model for studying the genetic basis of OED^16^. To test the role of endogenous GPR68 expression in susceptibility to chemical-induced oral dysplasia, age-matched WT (GPR68^+/+^) and KO (GPR68^−/−^) mice were placed on drinking water containing 4NQO (0.05 mg/mL) for 11–13 weeks, followed by normal water for 11–12 weeks. Mice gained weight throughout the period of 4NQO treatment, after which their weight plateaued and then slightly decreased by the end of the experiment (Fig. 1A). The maximum weight gain was approximately at week 16, reaching an average of 35% for WT males and 23% for WT females. This difference in body weight relative to week 0 was maintained until week 22, with WT males having gained significantly more weight compared to WT females (Fig. 1B). This was not due to differences in water consumption, as mice in each treatment group consumed approximately 6 mL of water per day throughout the first 11–13 weeks (Fig. 1C). GPR68 deficiency had no significant effect on body weight, as determined by an area under the curve analysis (Table 1). There was a trend towards decreased survival in KO mice, with 9% succumbing during the period of 4NQO treatment (Fig. 1D). This trend, however, was not statistically different than WT mice (*P* = 0.67), which had a similar 10% attrition rate at the end of the experiment. Overall, GPR68^−/−^ (KO) mice had normal weight gain, water consumption and survival following oral 4NQO treatment.

**Table 1:** Body weight area under the curve analysis of surviving mice.

Group (n)	Area under curve (body weight)	SEM	*P*-value relative to WTM
WT M (n = 9)	2837.0	107.5	n.a
WT F (n = 10)	2616.2	64.7	*P* = 0.09
KO M (n = 11)	2681.4	59.2	*P* = 0.20
KO F (n = 9)	2579.3	62.1	*P* = 0.054

**Figure 2:**
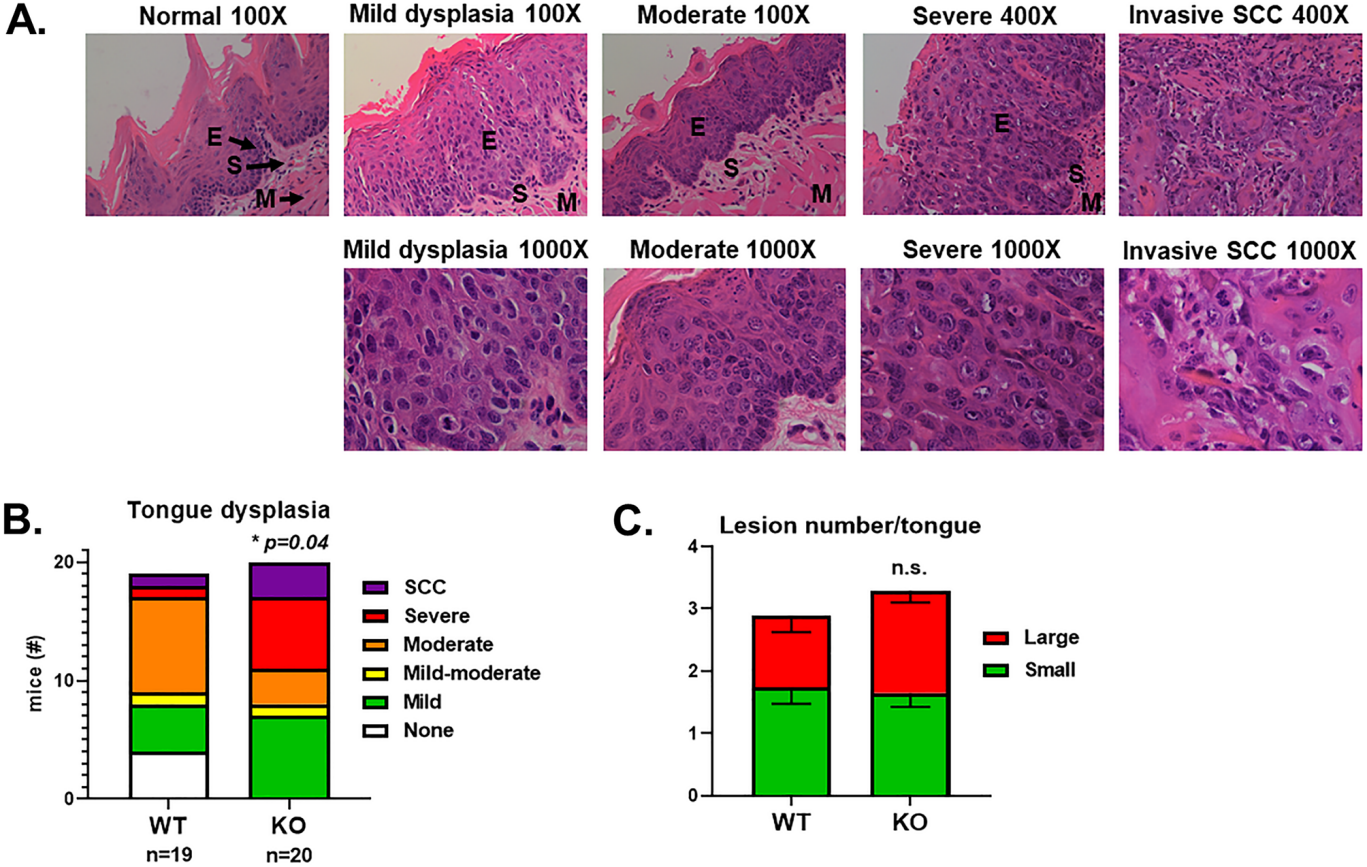
GPR68 deficiency increases the severity of 4NQO-induced tongue dysplasia. (**A**) Representative H&E-stained squamous mucosa with varying degrees of dysplasia on week 22 or 24. Mild dysplasia is characterized by changes confined to the lower one-third of the mucosa. Moderate and severe dysplasia have progressive involvement of the lower two-thirds to the upper one-third of the mucosa. Invasive squamous cell carcinoma has full thickness severe dysplasia with stromal invasion and permeation of malignant cells beyond the basement membrane. E = epithelium, S = stroma, M = muscle. The magnification for each panel is indicated. (B)** S**tacked chart showing the number of WT (left) or KO (right) mice with each grade of dysplasia. Statistical analysis was performed using a Chi-square test. (**C**) Stacked chart showing the mean (+/−SEM) number of large (red) or small (green) lesions per tongue on week 22 or 24. Statistical analysis was performed using Student’s t-tests for large and small lesions. Data are from the same experiments described in Fig. 1.

Haematoxylin and eosin staining is the gold standard for assessing potentially malignant lesions^17^. The severity of tongue dysplasia is determined by cytological and architectural changes that progressively involve greater regions of the epithelial layer (Fig. 2A). Overall, KO mice had an increased prevalence of severe dysplasia or SCC (Fig. 2B). Severe dysplasia shows abnormal proliferation into the upper third or full thickness of squamous mucosa, with prominent cytological and architectural changes, including pleomorphism, multiple nucleoli, apoptotic bodies and loss of architectural stratification^17^. Carcinoma in situ is the most severe form of OED which includes permeation of the basement membrane. Pathological examination revealed that 45 percent (9/20) of KO mice had either severe dysplasia or squamous cell carcinoma, compared to only 10.5 percent (2/19) of WT mice. This was associated with an increased percent of WT mice having moderate dysplasia (42% vs. 15%) or no dysplasia (21% versus 0%) compared to KO. Moderate dysplasia involves the proliferation of atypical cells in the lower two-thirds of the epithelium, with cytological characteristics including hyperchromatism, nuclear polymorphism and abnormal mitoses^17^. A Chi-square test revealed a statistically significant difference in dysplasia severity between WT and KO mice (*P* = 0.04; Fig. 2B). Macroscopic examination of tongues revealed that the total number and size of lesions was similar between WT and KO mice (Fig. 2C, Supplementary Fig. S1). These data suggest that GPR68 expression protects against oral tumorigenesis by limiting the severity of dysplasia.

### GPR68-deficiency did not affect angiogenesis within dysplastic oral lesions

**Figure 3:**
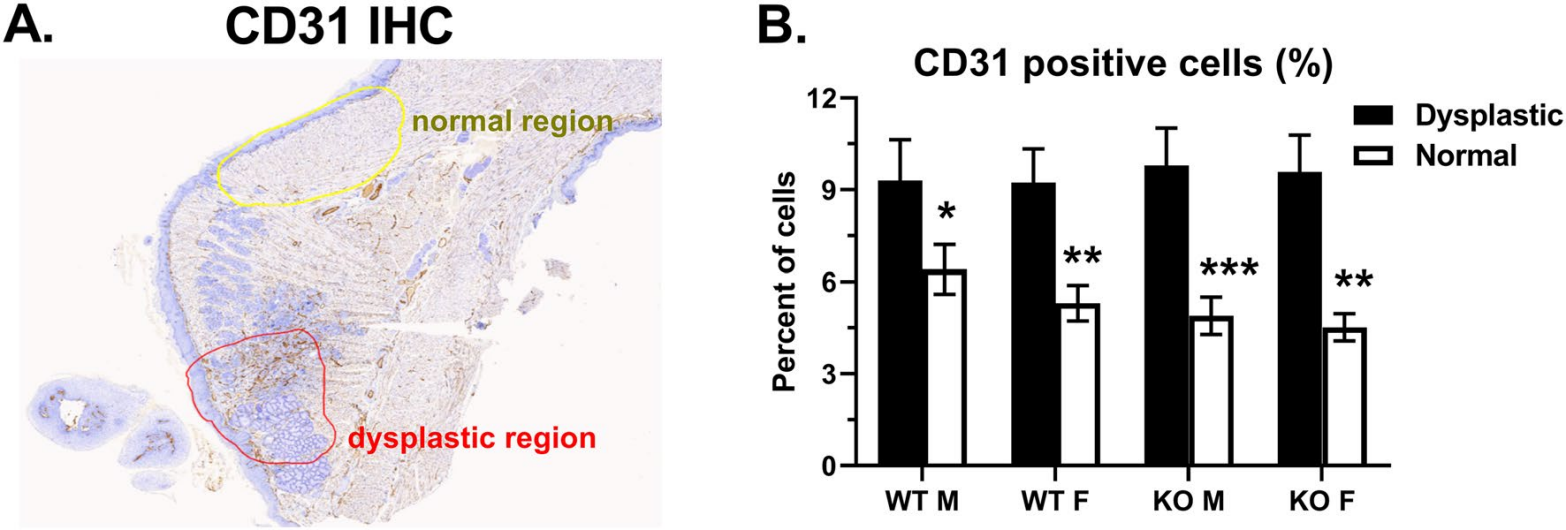
Dysplastic regions have increased CD31 expression independently of GPR68. Tongue sections from week 22–24 were stained for CD31 by immunohistochemistry (IHC). (**A**) Representative stain delineating the dysplastic region (red outline) from normal region (yellow). (**B**) Percent of cells staining positive for CD31 within the dysplastic (closed bar) or normal (open bar) regions. Data are from the same experiments described in Fig. 1 (*P < 0.05; **P < 0.01; ***P < 0.001).

Inducing angiogenesis is one of the six original hallmarks of cancer that enables tumor growth and metastatic dissemination, and occurs early during tumorigenesis^6^. To determine if GPR68 regulates angiogenesis in vivo, tongue sections were stained for CD31 by immunohistochemistry. Normal and dysplastic regions were analyzed separately to determine if dysplasia impacts the density of CD31 expression (Fig. 3A). In normal regions, approximately 5–6 percent of cells stained positive for CD31, with no significant differences between WT and KO strains (Fig. 3B). Dysplastic regions had significantly increased percentages of cells staining for CD31. The absolute percent (9–10%) was similar for both strains and genders analyzed, demonstrating that GPR68 did not regulate angiogenesis in response to 4NQO treatment (Fig. 3B).

### GPR68 alters the frequency of splenic regulatory T cells (Tregs) following oral 4NQO treatment

**Figure 4:**
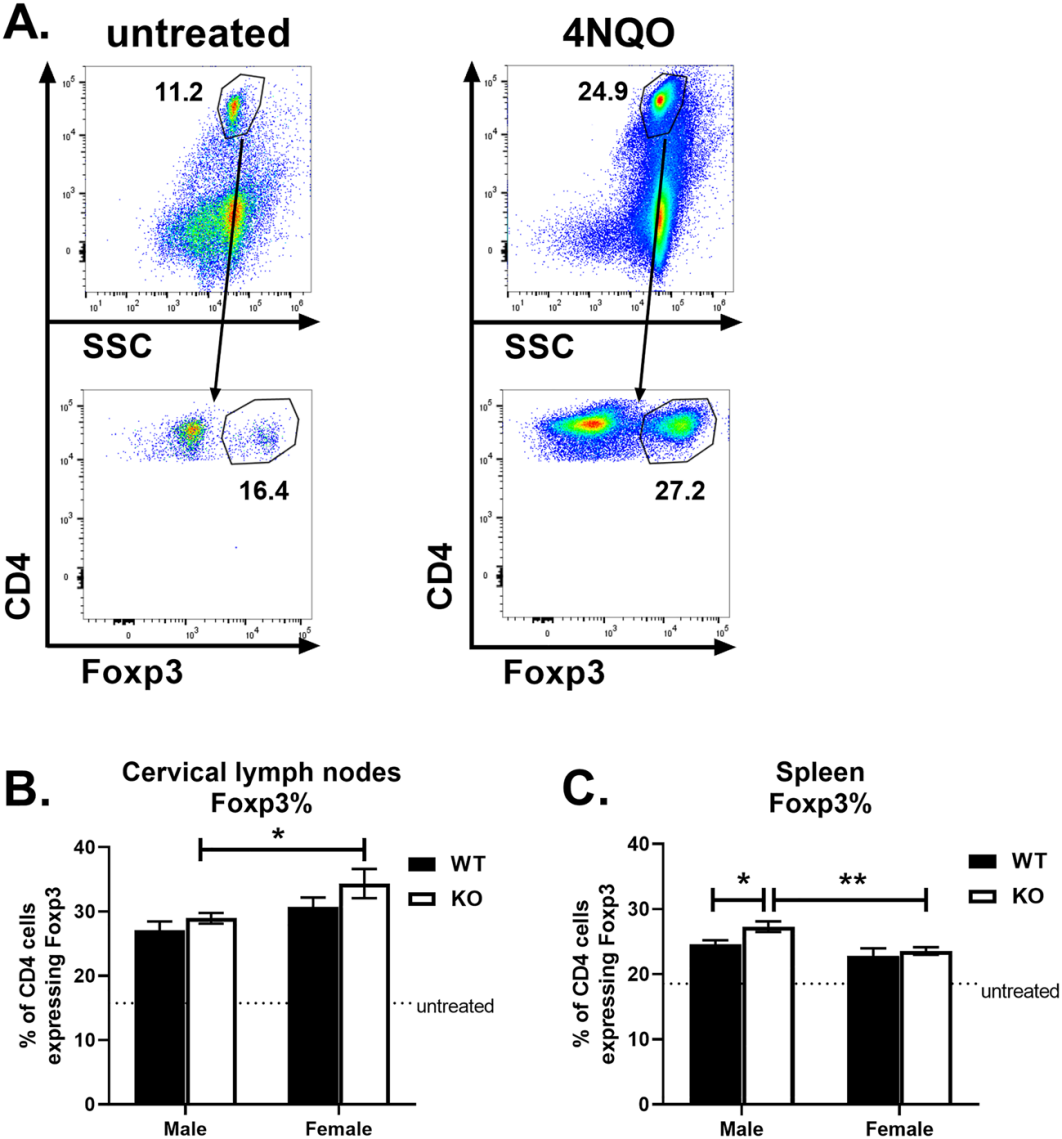
Role of GPR68 on Treg frequencies following 4NQO treatment. Single cell suspensions from cervical lymph nodes and spleen were stained for CD4 and Foxp3, and analyzed by flow cytometry. (**A**) Representative dot plots showing SSC vs. CD4 (top) and Foxp3 versus CD4 (bottom) from cervical lymph nodes of untreated and 4NQO-treated mice on week 24. (**B**) Mean +/− SEM of the percent of CD4 T cells expressing Foxp3 in cervical lymph nodes of WT (closed symbols) and KO mice (open symbols), as indicated. The dotted line represents Foxp3 frequencies in age-matched untreated mice. (**C**) Mean + /- SEM of the percent of CD4 T cells expressing Foxp3 in spleen of WT and KO mice, as indicated. Data are from the same experiments described in Fig. 1.

The mechanism through which GPR68 influences OED in vivo could be multi-factorial. Avoiding immune destruction is an emerging hallmark of cancer^6^, and regulatory T cells (Tregs) have been shown to suppress anticancer immunity^18^. To determine if GPR68 regulates Treg accumulation, cervical lymph node and spleen cells were stained for CD4 and Foxp3, and analyzed by flow cytometry. In untreated mice, the percent of CD4 T cells expressing Foxp3 was 15.8% in cervical lymph nodes and 18.6% in spleen (Fig. 4), and no significant differences were found between WT and KO mice (data not shown). 4NQO treatment increased Treg frequencies in WT male mice to 27.1% in lymph nodes and 24.6% in spleen, demonstrating that 4NQO regulates the balance of effector T cells and Tregs. GPR68 deficiency had no significant effect on Treg frequencies in cervical lymph nodes; however, male KO mice had significantly increased splenic Tregs (27.3%) compared to other groups (Fig. 4C), suggesting a greater immunosuppressive population was present.

**Figure 5:**
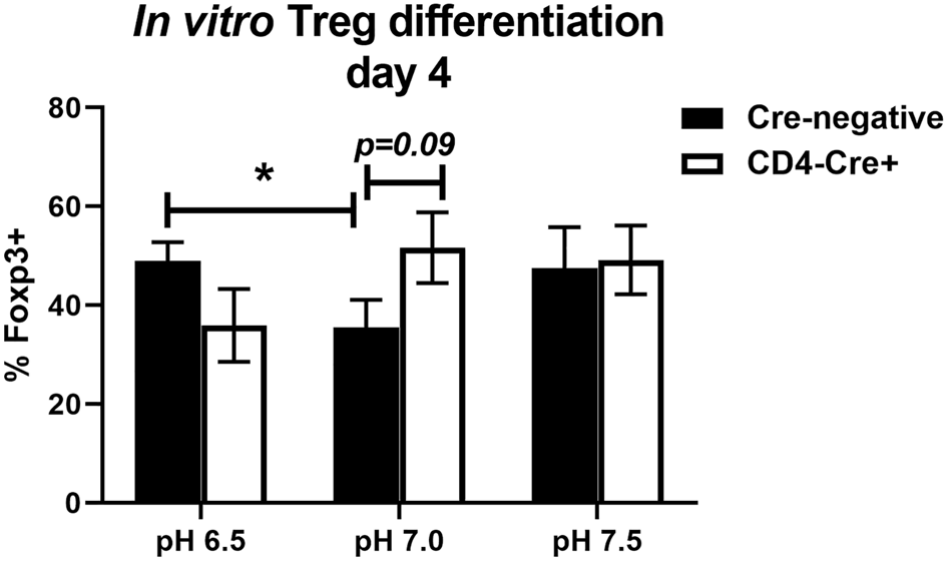
GPR68 does not intrinsically regulate Treg differentiation. Naïve CD4 T cells from GPR68^fl/fl^ (Cre-negative) and GPR68^fl/fl^ x CD4-Cre (CD4-Cre +) mice were cultured at pH 6.5, 7.0 and 7.5 with anti-CD3/CD28 beads (1:3 bead:cell ratio), IL-2 (10 ng/mL) and TGF-b (5 ng/mL). On day 4, cells were stained with CD4 and Foxp3, and analyzed by flow cytometry. The percent of CD4 T cells expressing Foxp3 is shown as the mean + /- SEM. Data are combined from four experiments with n = 13–17.

To determine if GPR68 expression in T cells suppresses Treg differentiation, naïve CD4 T cells were purified from spleens of age-matched GPR68^fl/fl^ and GPR68^fl/fl^ × CD4-Cre^+^ mice, and cultured with CD3/CD28 stimulation, TGF-β and IL-2 for four days. In these mice, expression of Cre recombinase under the CD4 promoter results in genetic deletion of GPR68. To determine the impact of extracellular pH on Treg differentiation, Cre^+^ (KO) and Cre^−^ (WT) cells were cultured at pH 6.5, 7.0 and 7.5. WT CD4 T cells cultured at pH 7.0 had significantly fewer cells expressing Foxp3 compared to pH 6.5, suggesting that extracellular pH may regulate Treg differentiation (Fig. 5). No significant difference was observed between WT and KO T cells at pH 6.5, 7.0, or 7.5, demonstrating that GPR68 did not impact Treg differentiation in a T cell-intrinsic manner.

### Transfection with GPR68-specific siRNA did not affect cell survival or proliferation in vitro

**Figure 6:**
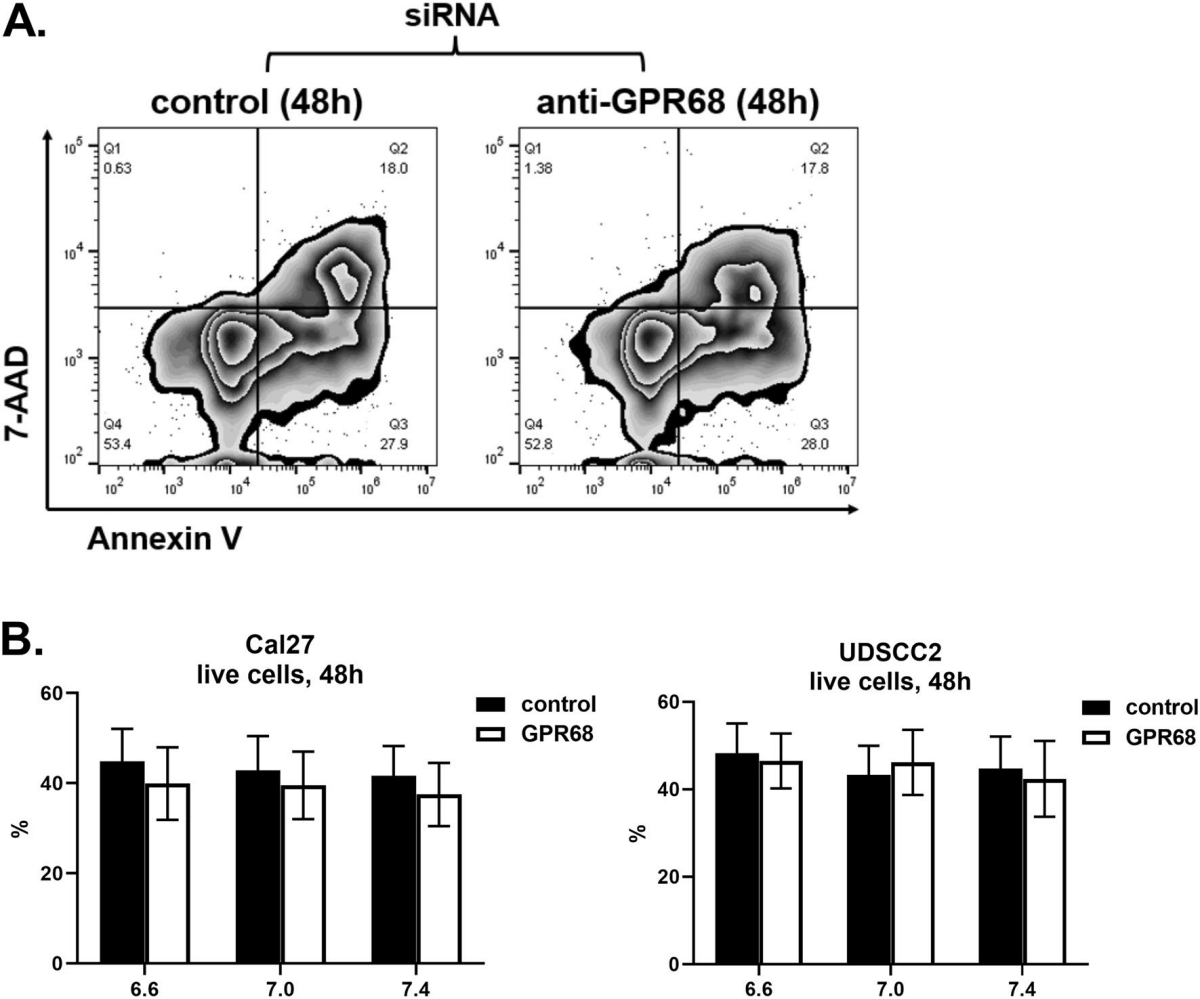
GPR68 knockdown had no significant effect on cell line survival. Cal-27 and UD-SCC-2 cells were cultured at pH 6.6, 7.0 and 7.4 with siRNA specific for GPR68 (open bars) or a negative control (closed bars), as indicated. At 48 h, cells were scraped, stained for Annexin V and 7-AAD, and analyzed by flow cytometry. (**A**) Representative flow cytometry plots showing Annexin V vs. 7-AAD staining. (**B**) Charts showing the percent of live double-negative (Annexin V^-^ 7-AAD^-^) cells as mean +/− SEM. Data are combined from 6 experiments with n = 16–18 (Cal-27), or from three experiments with n = 9 (UD-SCC-2).

**Figure 7:**
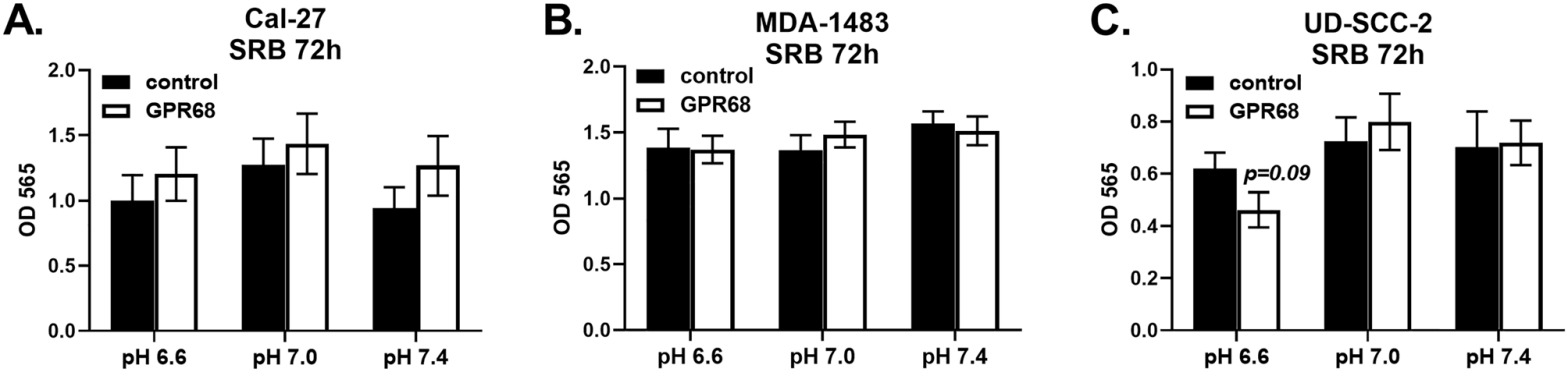
GPR68 knockdown had no significant effect on cell line growth. Cal-27 (**A**), MDA-1483 (**B**) and UD-SCC-2 (**C**) cells were cultured for 72 h with siRNA specific for GPR68 (open bars) or a negative control (closed bars), as indicated. An SRB assay was used to measure cell growth, with data reported as the mean +/− SEM of the optical density at 565 nm. Data are combined from 4 experiments with n = 16 (**A**), 3 experiments with n = 14 (**B**) or 3 experiments with n = 12 (**C**).

To determine if GPR68 gene expression is regulated by pH, several HNSCC cell lines (Cal-27, FaDu, JHU-022, MDA-1483, UD-SCC-2, UM-SCC-47, UPCI-SCC-090) were cultured at pH 6.6, 7.0 and 7.4 for three days. Each of the cell lines expressed GPR68 at comparable levels by PCR; however, extracellular pH did not significantly affect GPR68 expression at these time points (data not shown). We next wanted to determine if GPR68 regulates cancer cell growth or survival, as several hallmarks of cancer impact these processes^6^. Towards this, Cal-27 and UD-SCC-2 cell lines were transfected with siRNA targeting GPR68, or a negative control, and stained for Annexin V and 7-AAD. Extracellular pH had no significant effect on cell survival at 48 h (Fig. 6), suggesting that proton-sensing receptors may not regulate apoptosis. Consistent with this, no difference was observed in Annexin V staining between cells transfected with GPR68-specific or control siRNA. Sulforhodamine B (SRB) assays were used to assess the impact of GPR68 knockdown on growth of Cal-27, MDA-1483 and UD-SCC-2 cell lines. We find that extracellular pH and siRNA targeting GPR68 had no significant effect on cell growth (Fig. 7). Overall, our data suggests that GPR68 expression protects against the severity of chemical-induced oral dysplasia in a tumor cell-extrinsic manner.

## Discussion

Elucidating the pathophysiological mechanisms of OED development provides opportunities for therapeutic intervention. Cancer cells have an altered metabolic profile that favors aerobic glycolysis, resulting in extracellular acidification^7^. In addition, the poor vascularization of tumors leads to microenvironmental gradients in metabolites and oxygen, further promoting the accumulation of lactate and hydrogen ions^19^. This results in the local activation of proton-sensing receptors that may regulate several aspects of tumorigenesis including proliferation, autophagy, metastasis, angiogenesis and immune cell function^9^. Previously, a gene expression analysis revealed that GPR68 is upregulated in head and neck cancer and associated with cisplatin-resistance, hypoxia, angiogenesis and TGF-β expression^20^. Here, using a murine model of chemical-induced OED, we showed that GPR68-deficiency increases the incidence of severe dysplasia and SCC (Fig. 2B), suggesting that endogenous GPR68 may attenuate cancer progression.

The incidence of severe OED and SCC that we observed was lower than has previously been reported in studies that used higher doses or longer durations of 4NQO treatment^21–24^. These studies reported an incidence of severe dysplasia or SCC of 57–100%. In comparison, our model was less pathogenic for WT mice, allowing us to study mouse strains with increased susceptibility to OED. Most of the mice with severe dysplasia or SCC in our study were cohoused with others classified as having mild or moderate dysplasia, demonstrating intragroup variation among those sharing the same water. The significantly increased prevalence of severe dysplasia or SCC in tongues of GPR68^−/−^ mice suggests a potential role for this gene in suppressing the initiation or progression of OED.

Male GPR68^−/−^ mice had significantly increased frequencies of splenic Tregs compared to their GPR68^+/+^ counterparts (Fig. 4C). This is interesting, as patients with HNSCC also have elevated peripheral levels of Tregs^25^. Treg accumulation may be one of the mechanisms through which SCC suppresses immunosurveillance, although recent studies found that high Treg density in oropharyngeal SCC tumors or draining lymph nodes correlate with better prognosis^26,27^. A rat model recapitulated the human findings in which 4NQO increases Foxp3^+^ cells in peripheral blood and cervical lymph nodes, correlating with disease severity^28^. Further, a murine model reported increased Foxp3 expression in tongues 20 weeks following 4NQO treatment^29^. The mechanism through which GPR68 affects Treg accumulation in vivo could be due to its intrinsic expression in CD4 T cells, or extrinsic expression in non-T cells. Our in vitro experiments indicated that GPR68 expression in CD4 T cells did not influence Treg differentiation (Fig. 5). It remains to be determined if extracellular acidification impacts the expression of chemokines required for Treg recruitment (CCL1, CCL5, CCL22, CCL28, CXCL12) or the signaling pathways required for Treg differentiation (STAT5, NFAT, PI3K, AKT, mTOR)^18^. Given the link to Treg elevation in clinical cases, future studies are necessary to determine how the tumor microenvironment, including GPR68 expression, regulates their accumulation and function.

The wide tissue distribution of GPR68 suggests it may regulate several of the defined hallmarks of cancer^6^. To determine if GPR68 influences cell growth or survival in vitro, head and neck cancer cell lines were treated with siRNA at pH 6.6, 7.0 and 7.4. We found no significant effect of GPR68 knockdown on sulforhodamine B or Annexin V staining, suggesting that GPR68 knockdown does not lead to apoptosis in mild acidosis. Cellular stress contributes to the pathology associated with 4NQO treatment. A previous study found that GPR68 deletion in the brain reduces the expression of several proteins important for ER stress responses including GRP78/BiP and HSP70^30^. We speculate the GPR68 deletion during 4NQO treatment may exacerbate tongue injury due to cellular stress, aligning with the observation of severe dysplasia observed in GPR68^−/−^ mice.

GPR68 can suppress angiogenesis in acidic media, as measured by tube formation^31^. We did not observe any significant differences between GPR68^+/+^ and GPR68^−/−^ mice in CD31 expression within dysplastic tissue (Fig. 3), suggesting that GPR68 did not regulate angiogenesis in vivo. GPR4 has been shown to induce angiogenesis in response to acidification by promoting the expression of IL-6, IL-8 and VEGF^32^. Further, renal cell carcinomas and epithelial ovarian carcinomas showed a correlation between GPR4, VEGFA and microvascular density^33,34^. Murine models also demonstrated a critical role for GPR4 in promoting angiogenesis following tumor injection or colitis induction^35,36^. Overall data suggests that GPR4 promotes angiogenesis due to its high expression within vascular endothelial cells. In addition, GPR4 has been suggested to be an oncogene in an ovarian cancer cell line^37^. Further studies are necessary to delineate cell type-specific roles for other proton-sensing receptors in cancer.

The immune system has an integral role in tumor immunosurveillance, with Tregs, myeloid-derived suppressor cells (MDSCs), and tumor-associated macrophages being implicated in tumor immune escape. In addition, tumor cells can upregulate CTLA-4, PD-1 or PD-L1 to suppress immune cells. Further studies are necessary to determine if GPR68 regulates the function of MDSCs or macrophages in the context of tumorigenesis, or if GPR68-deficient tumors have elevated expression of inhibitory receptors. A previous study demonstrated that macrophages with GPR68 deficiency are more potent than wild-type macrophages at suppressing tumor growth, correlating with iNOS expression^13^. Further, low pH upregulated PD-L1 in murine squamous cell carcinoma and melanoma cell lines in a GPR68- and GPR65-dependent manner^38^. Neutralizing tumor acidity with sodium bicarbonate reduced PD-L1 expression and tumor growth in vivo, demonstrating a possible role for proton-sensing receptors in immune escape. Other studies have shown that GPR68 expression in non-tumor cells can promote carcinogenesis. Transplanting cancerous cells into GPR68^−/−^ mice reduced tumor development through augmented function of cytotoxic CD8 T cells and macrophages^12,13,39,40^. These data demonstrate multiple mechanisms through which GPR68 can regulate the severity of tumorigenesis through immunosurveillance. The complex roles for GPR68 may be attributed to the tumor type, stage of cancer and role of non-tumor cells (stromal, endothelial, immune).

Proton-sensing receptors have been shown to augment chronic inflammation in various models. GPR68 is overexpressed in fibrotic intestinal regions in humans with IBD, and genetic deletion or suppression of GPR68 attenuates intestinal inflammation^41–43^. Likewise, GPR4 has a pro-inflammatory role in IBD^44–46^. GPR65-deficient mice had a mild exacerbation of tumorigenesis in a model of colitis-associated colorectal cancer^47^, suggesting that GPR65 has anti-tumor functions in the GI tract. A murine model of multiple sclerosis, experimental autoimmune encephalomyelitis, demonstrated a pathogenic role for GPR68 in promoting disease^48^. This was associated with reduced numbers of Th1 cells, Th17 cells, macrophages and dendritic cells in GPR68^−/−^ mice following immunization. In an asthma model, GPR68^−/−^ mice on a BALB/c background had reduced airway hyperresponsiveness, inflammation and Th2 cytokines following sensitization and challenge with ovalbumin^49^. This was associated with a reduced ability of CD11c^+^ dendritic cells from GPR68^−/−^ mice to transfer airway hyperresponsiveness to WT mice. Antagonists of OGR1 and GPR4 given systemically have shown anti-inflammatory and anti-fibrotic actions in mice^35,41,45^. In airway smooth muscle cells, acidification induces CXCL8 and connective tissue growth factor in a GPR68-dependent manner^50,51^. Overall, modulating inflammation may be one of the mechanisms through which GPR68 regulates susceptibility to chronic diseases.

Although recent developments in molecular-targeted therapies, including immune-based drugs, are rapidly changing the landscape of cancer therapeutics, treating HNSCC remains challenging. Immunotherapy regimens targeting the PD-1 or EGFR pathways have variable response rates^52,53^, demonstrating the importance of identifying novel molecular pathways associated with HNSCC carcinogenesis. For instance, the efficacy of anti-PD-1 therapy is limited to patients with high PD-L1 expression, which is heterogeneously expressed in tumors^54^. Here, we report a provocative finding for GPR68 in limiting the severity of chemical-induced OED, correlating with increased frequencies of splenic Tregs in male GPR68^−/−^ mice. Future studies are necessary to test if GPR68 agonists, or modulators of other proton-sensing receptors, help to uncouple cellular energetics from carcinogenesis. Notably, suppressing aerobic glycolysis with 2-deoxy-D-glucose enhanced the anti-tumor efficacy of zoptarelin doxorubicin in a breast cancer cell line^52^, demonstrating synergism. GPR68 potentiation may provide one potential anti-cancer strategy and will be important to assess in a future study.

## Methods

### Mice

GPR68^−/−^ and GPR68^fl/fl^ mice on the C57BL/6 background were generated as described^12^. Heterozygous (GPR68^+/−^) breeders were used to produce the wild-type (WT; GPR68^+/+^) and knockout (KO; GPR68^−/−^) strains in-house. GPR68^fl/fl^ mice were crossed to CD4-Cre (Jackson Labs 022,071) to generate a strain with GPR68 deleted in T cells. Male GPR68^fl/fl^ × CD4-Cre^+^ mice bred with female GPR68^fl/fl^ mice to generate Cre^+^ and Cre-negative littermates. Mice were genotyped by PCR analysis of tail DNA. All experiments were performed in accordance with relevant institutional and national guidelines, regulations, approvals and in compliance with the ARRIVE (Animal Research: Reporting of In Vivo Experiments) guidelines. All experiments involving animals were approved by the Institutional Animal care and Use Committee (IACUC) and Institutional Biosafety Committee of Marshall University.

### Mouse treatments

Six- to eight-week old control (GPR68^+/+^; WT) and GPR68-deficient (GPR68^−/−^; KO) mice were placed on drinking water containing 4-Nitroquinoline N-oxide (4NQO; 0.05 mg/mL; Millipore Sigma; St. Louis, MO) for 11–13 weeks, followed by normal water for 11–12 weeks. Body weight and water consumption were measured weekly, with tissues collected on week 22 or 24.

### Tissue processing

Tongues were dissected and placed in Z-Fix (Anatech Ltd; Battle Creek, MI), followed by 70 percent ethanol. Prior to fixing, the number of lesions/tongue were recorded and categorized as large or small. Fixed tongues were paraffin-embedded, sectioned and stained with hematoxylin and eosin (H&E) or CD31 (iHisto, Inc; Salem, MA). Cervical lymph nodes and spleens were crushed through 100uM cell strainers, rinsed with balanced salt solution and stained for CD4-fluorescein isothiocyanate (clone GK1.5; Biolegend, San Diego, CA) and Foxp3-phycoerythrin (clone FJK-16S; ThermoFisher Scientific, Walthan, MA). Data was collected on a Novocyte 2000R flow cytometer (ACEA Biosciences; San Diego, CA) and analyzed using FlowJo (Ashland, OR) software.

### Hematoxylin and Eosin (H&E) analysis

The H&E slides were reviewed in a blinded fashion by a single pathologist (V.G.) and assessed for evidence of squamous dysplasia or invasive carcinoma. A three tier system for classifying and grading squamous dysplasia was applied: mild, moderate and severe dysplasia^55^. Dysplastic features in squamous cells include nuclear pleomorphism and hyperchromasia with loss of normal cell polarity within the mucosa. Dysplastic features confined to the lower one-third of the mucosa were classified as mild dysplasia, the lower two-thirds moderate dysplasia and severe dysplasia where the changes involved the upper-third or full thickness of the squamous mucosa. Invasive squamous cell carcinoma was characterized by severe dysplastic changes with permeation of the basement membrane.

### Immunohistochemistry

Slides were stained with an anti-mouse CD31 rabbit monoclonal antibody (clone D8V9E; Cell Signaling Technology), detected with DAB, scanned with a 40X objective (iHisto, Inc), and analyzed using Qupath 0.3.2 software. Dysplastic and normal regions of each classified sample were separately annotated by Qupath’s brush tool and independently measured for CD31 expression. The percent of CD31-positive cells was determined using a threshold “full” resolution at 0.26 um/px. The DAB channel was used with a Gaussian pre-filter. Each threshold was set at 0.1 with pixels above 0.1 being positive for CD31 and pixels below 0.1 being negative. Percentages and surface area of each classifier was noted for the dysplastic and normal region, and graphed for patterns.

### Treg differentiation

Naïve CD4 T cells were purified from spleens and lymph nodes of GPR68^fl/fl^ (Cre-negative) and GPR68^fl/fl^ x CD4-Cre (CD4-Cre^+^) mice, and cultured in flat-bottom 96-well plates at 1.2–1.6 million cells/mL for four days in Iscove’s Modified Dulbecco’s Medium (Gibco) supplemented with 10% fetal bovine serum, anti-CD3/CD28 dynabeads (1:3 bead:cell ratio; ThermoFisher), IL-2 (10 ng/mL; R&D Systems), and TGF-b (5 ng/mL; R&D Systems). Media pH was adjusted to 6.5, 7.0 or 7.5 using hydrochloric acid or sodium hydroxide. On day 4, cells were stained with CD4-APC (Biolegend) and Foxp3 (ThermoFisher), and analyzed by flow cytometry on a Novocyte 2000R (ACEA Biosciences).

### Cell culture

Head and neck squamous carcinoma cell lines (MDA-1483, UD-SCC-2, Cal-27, FaDu, UM-SCC-47, UPCI-SCC-090, JHU-022) were cultured in DMEM/F-12 (1:1) (Gibco) supplemented with 10% fetal bovine serum and penicillin/streptomycin. For experiments, cells were trypsinized and plated for 2–3 days, with the media pH adjusted using hydrochloric acid and sodium hydroxide. GPR68 expression was silenced with siRNA (Ambion 4,392,420) and compared to a negative control (Ambion 4,390,843). Briefly, the siRNA was mixed with Lipofectamine RNAiMAX (Invitrogen) according to the manufacturer’s instructions and added to cells. For detecting apoptosis, 1 × 10^5^ cells were added to 24-well plates (Corning Costar). The next day, media was replaced with fresh media adjusted to pH 6.6, 7.0 or 7.4 using hydrochloric acid or sodium hydroxide, and treated with siRNA. 48 h later, cells were scraped, stained with Annexin V-phycoerythrin and 7-AAD (Biolegend), and analyzed by flow cytometry on a Novocyte 2000R. Cell growth was measured using a sulforhodamine B assay (SRB; BioVision, Inc., San Francisco, CA). Briefly, 5 × 10^3^ cells were added to 96-well plates (Fisher Scientific). The next day, media was replaced with fresh media adjusted to pH 6.6, 7.0 or 7.4, and treated with siRNA. 72 h later, cells were fixed, stained and analyzed at 565 nm using a Multiskan GO (ThermoFisher Scientific).

## Supplementary Information

Supplementary Information.

## Data Availability

All data generated or analyzed during this study are included in this published article and its supplementary information files.
