# Supplementary material for: GPR68 limits the severity of chemical-induced oral epithelial dysplasia
Source: Sci Rep. 2023 Jan 7;13:353. doi: 10.1038/s41598-023-27546-y (PMC9825365; doi:10.1038/s41598-023-27546-y)
Supplement: Supplementary file 1 — Supplementary Information. [file 41598_2023_27546_MOESM1_ESM.pdf]

GPR68 Limits the Severity of Chemical-Induced Oral Epithelial Dysplasia

David Shore[1], Nosakhere Griggs[1], Vincent Graffeo[2], ARM Ruhul Amin[1], Xiang-ming Zha[3], Yan Xu[4], and Jeremy P. McAleer[1*]

[1]Marshall University School of Pharmacy, Huntington, WV; [2]Marshall University Joan C. Edwards School of Medicine, Huntington, WV; [3]University of Missouri-Kansas City School of Pharmacy, Kansas City, MO; [4]Indiana University School of Medicine, Indianapolis, IN

*Corresponding author: mcaleer@marshall.edu

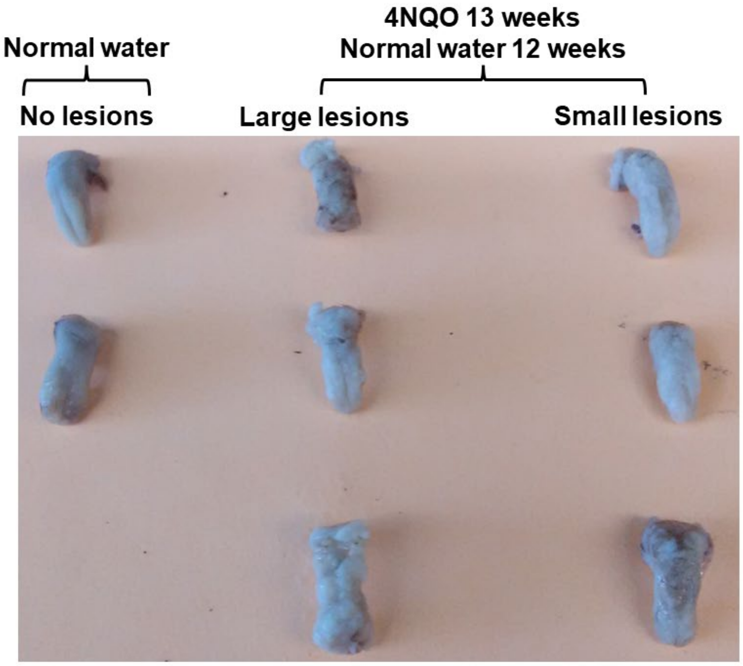

Supplementary Figure S1. Macroscopic images of tongue lesions. Representative tongues for analysis of superficial lesions. The lesions were classified as small or large based on their relative surface area of the dorsal tongue.
